# Pancreaticopleural Fistula as a Rare Cause of Both-Sided Pleural Effusion

**DOI:** 10.1155/2021/6615612

**Published:** 2021-03-02

**Authors:** Jan Chmielecki, Tomasz Kościński, Tomasz Banasiewicz

**Affiliations:** Department of General, Endocrinological and Gastroenterological Surgery, Poznań University of Medical Sciences, Poland

## Abstract

A pancreaticopleural fistula is a rare cause of pleural effusion. It is a complication of chronic or acute pancreatitis. It is rarely formed to the right or both pleural cavities. Diagnosis and proper treatment often turn out to be difficult and require the cooperation of a multidisciplinary team. The authors present the case of a 59-year-old patient treated for recurrent pleural effusion of unknown origin, first to the left and then to the right pleural cavity. After many months of treatment, the diagnosis of a pancreaticopleural fistula was made. The patient underwent surgery, which finally led to a successful complete recovery. Pancreaticopleural fistula should always be considered in patients with pleural effusion of unknown origin.

## 1. Introduction

Pancreatic fistula is an uncommon complication of acute or chronic pancreatitis. It develops as a result of pseudocyst rupture or pancreatic duct perforation. Most often, pancreatic secretions enter the peritoneal cavity and cause pancreatic ascites. Pancreaticopleural fistula formation is a very rare complication of chronic pancreatitis and even more rarely seen in acute pancreatitis [1, 2]. Determining the true frequency of this complication is difficult due to the challenges associated with making the diagnosis and, consequently, the resulting treatment errors [3–5]. The most common location for a pancreatic fistula is to the left pleura. Fistulas to the right or both pleural cavities are much less common [6]. The authors present a case of a patient with a chronic pleural effusion, who went through a very long diagnostic process, and finally was diagnosed with pancreaticopleural fistula first to the left and then to the right pleural cavity. The patient underwent surgery, which eventually led to a complete recovery.

## 2. Clinical Features, Diagnosis, and Treatment

We present a 59-year-old man, admitted to the Department of General, Endocrinological, and Gastroenterological Surgery in Poznań in September 2019 with chronic pancreatitis and suspicion of a pancreaticopleural fistula. The patient was hypertensive but denied smoking or alcohol use. Past medical history showed that the patient was healthy, quite athletic with no diabetes, tuberculosis, or chronic lung disease. Before admission to our department, the patient was hospitalized many times over the course of a year and treated for pleural effusions of unknown origin.

**Figure 1:**
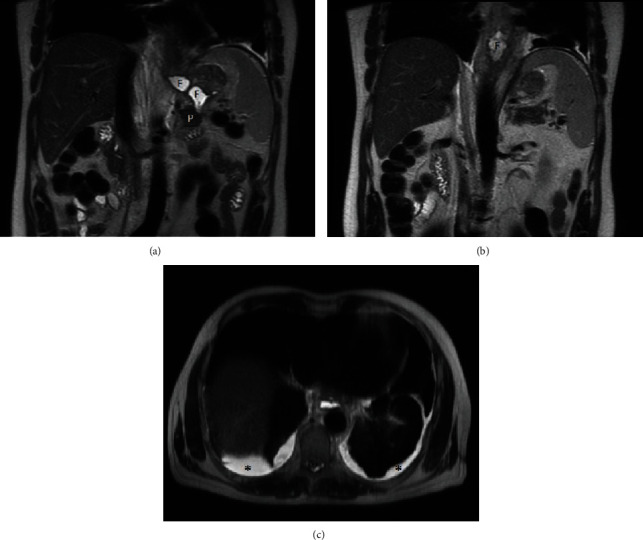
Magnetic resonance. Coronal and axial T2 single shot fast spin echo sequences revealed retroperitoneal peripancreatic fluid collections (a) extending to mediastinum (b) and free fluid in both pleural cavities (c). P: pancreas; F: fluid collection. ^∗^Free fluid in pleural cavity.

**Figure 2:**
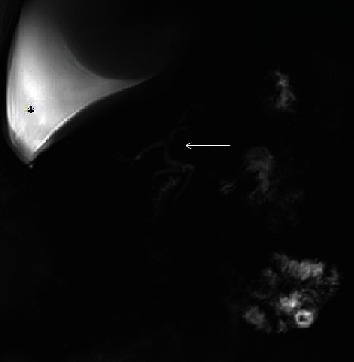
Magnetic resonance cholangiopancreatography. Coronal reconstruction. Normal caliber of bile and pancreatic ducts, no connection with fluid collections. Massive amount of free fluid in the right pleural cavity. White arrow: cholangiopancreatic ducts. ^∗^Free fluid in pleural cavity.

**Figure 3:**
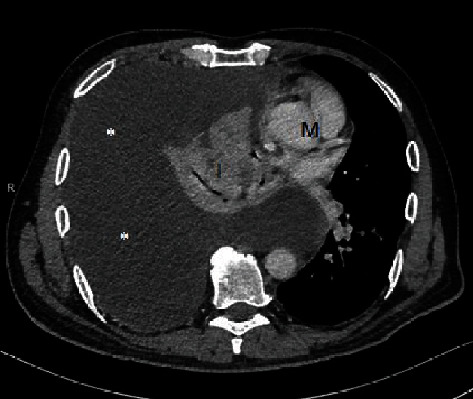
Computed tomography of chest cavity after intravenous contrast injection in axial plane. Free fluid in the right pleurum with atelectasis of the right lung and mediastinal shift. ^∗^Free fluid in pleural cavity. L: lung; M: mediastinum.

**Figure 4:**
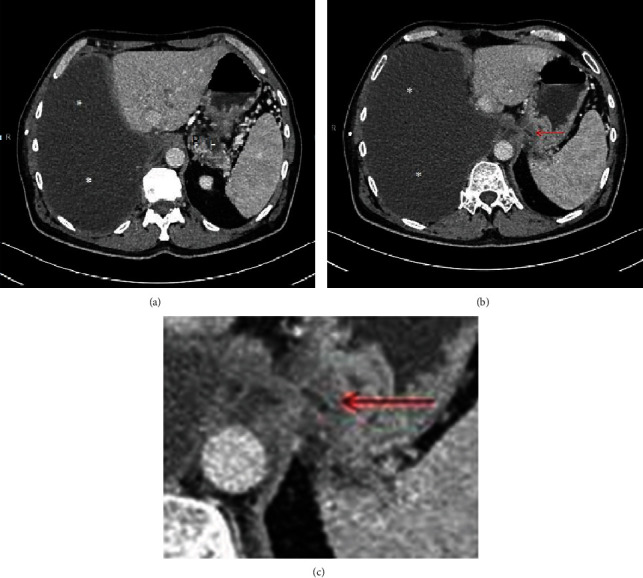
Computed tomography of the abdomen after intravenous contrast injection in axial plane. Enhancement of fistulous track walls that connect peripancreatic fluid collection with right pleural cavity (a, b). Fistula is better seen after enlargement (c). P: pancreas; F: fluid collection. ^∗^Free fluid in pleural cavity. Red arrow: fistula.

The patient's medical history began with a sudden onset of chest pain radiating to the back. He presented to his outpatient clinic and was referred for a thoracic spine computed tomography (CT). Accumulation of fluid in the left pleural cavity was identified. The patient was referred to the pulmonary ward, already presenting with cough, moderate dyspnea, and a tightness on the left side of the chest. CT scan of the thorax and abdomen was performed which revealed fluid in the left pleural cavity, left lung atelectasis, and asymmetric thickening of the lower esophagus up to 26 mm, mainly on the left side. A note was also made of a 10 mm hypodense lesion in the pancreas tail. Using endoscopic ultrasound (EUS), the esophageal lesion was suspected to represent a Gastrointestinal Stromal Tumor (GIST), and material for cytological testing was collected through an EUS biopsy. A left thoracentesis was performed, and 1600 ml of bloody fluid was evacuated and sent for further cytological and microbiological tests. Only methicillin-susceptible S. epidermidis grew in the pleural sample: the patient was discharged home in good condition. After a week, he was readmitted because of increasing dyspnea. Due to the significant amount of reaccumulated fluid in the left pleura, a Robinson drain was placed in the left thoracic space. The patient was also referred to a gastroenterological outpatient clinic to follow up on the suspected esophageal GIST. During another scheduled hospitalization, videoscopic decortication of the left lung was performed. Lung expansion was achieved, but repeat CT scan showed an increase in the hypodense lesion in the pancreas tail. Histopathological examination of the pleura did not reveal any atypical cells. The patient was discharged home. After a month, he presented to the emergency department because of abdominal pain. Acute pancreatitis was diagnosed, and the patient was admitted to the general surgery department. The patient's general condition and laboratory test results improved after the conservative treatment; therefore, the patient was discharged home with a pancreatic diet recommendation. In the outpatient clinic, an abdominal magnetic resonance imaging (MRI) (Figure 1) was performed. It demonstrated edema of the esophagus and pancreatic tail as well as several small fluid collections in the retroperitoneal space extending from the distal, swollen part of the esophagus to the pancreatic tail. There were also free fluid collections in the right pleural cavity and around the left lung. Shortly after this examination, the patient was again admitted to the pulmonology ward with acute shortness of breath and pain on the right side of the chest. Due to the large amount of fluid in the right pleural cavity, thoracentesis was performed, and the pleural fluid was tested for amylase. This laboratory test was performed for the first time. The level of the amylase was very high (17250 U/l). Serum and urine amylase results were also significantly increased. The patient was discharged home with a recommendation for further urgent gastroenterological diagnostics, although this ended up being delayed. After another few weeks, due to the shortness of breath, he presented to the emergency ward, and another right thoracentesis was performed. The date of planned right-sided pleurodesis was set during the thoracic surgery consultation. In the next few days, the patient presented to the gastroenterological outpatient clinic. The chest X-ray, serum and urine amylase, C-reactive protein (CRP), and magnetic resonance cholangiopancreatography (MRCP) (Figure 2) were performed. Due to high levels of amylase, a right hydrothorax, shortness of breath, and poor general condition, the patient was admitted to the Gastroenterological Department. MRCP showed normal diameters of the bile duct and pancreatic duct. There were no enlarged lymph nodes or retroperitoneal fluid. Chest CT (Figure 3) was performed, and a large amount of fluid in the right pleural cavity was seen once again. It was causing atelectasis of the right lung and 60 mm displacement of the mediastinal structures to the left, as well as compression of the left lung. Drainage of the right pleural cavity was performed. Lab tests showed high levels of amylase in the right pleural fluid. CT of the abdomen (Figure 4) was performed which demonstrated an 18 mm fluid collection next to the pancreas tail with an associated inflammatory infiltration of the retroperitoneal space, extending towards the aortic hiatus of the diaphragm and right pleural cavity. The patient was transferred to the Department of General, Endocrinological, and Gastroenterological Surgery for surgical intervention. At laparotomy, a peripancreatic tail collection as well as inflammatory infiltration of the retroperitoneal space connecting to the posterior wall of the gastric fundus and left adrenal gland was found. When the pancreatic collection was opened, the fistula channel was visible, running in the retroperitoneal space to the right pleura. Fistula dissection and distal pancreatic resection were performed. The pancreatic-side opening (originally connected to the Wirsung duct) of the fistula was oversewn. A splenectomy also was performed due to thrombosis of the splenic vein noted on the most recent CT scan. A drain was placed near the closed fistula. In the postoperative days, the patient's general condition improved, with reduction of the right pleural exudation and partial expansion of the right lung. Histopathology showed chronic inflammatory lesions of the pancreatic tail and the canal with fibrinous and purulent masses. The edema of the esophagus was the inflammatory reaction to the pancreaticopleural fistula (not GIST, as was initially suspected based on the CT scan). Due to incomplete expansion of the right lung and persistent exudate in the right pleura, the patient was transferred to the pulmonary and rehabilitation department. Finally, after successful rehabilitation, the patient was discharged home, remaining under observation for over a year, without any recurrent symptoms.

## 3. Discussion

Pancreatitis-related pleural effusions are rare complications of pancreatitis occurring in 3-7% of patients with pancreatitis. However, pleural effusion due to a pancreaticopleural fistula is very rare. Most authors rate the incidence of this complication as less than 1% [1–3, 7]. The main cause of these fistulae is a chronic inflammatory process of the pancreas, most often attributed to alcoholic pancreatitis [8]. The diagnosis of pancreaticopleural fistula is often extremely difficult, especially in patients without a history of pancreatitis and in those who do not have any abdominal symptoms. The delay in diagnosis usually ranges from 12 to 49 days [9]. Pancreaticopleural fistula is sometimes mistaken for other diseases. In this case, malignant disease of another nearby organ (esophagus) was initially suspected, but diagnostic mistakes for pleural tuberculosis and alcoholic liver disease were also previously reported [8, 10]. During this patient's many hospitalizations, the treating teams involved in the patient's care were focused on diagnosing the most common causes of his manifested symptoms, not considering the rarer ones. Nonspecific imaging appearances can also lead to delays in the final diagnosis [7, 11]. Pancreaticopleural effusion should always be taken into consideration in patients with hydrothorax of unknown origin resistant to standard therapy [12]. This diagnosis can be confirmed by a high amylase level in a pleural exudate, but it is not always a routine test to evaluate. The breakthrough in diagnosis in this patient was the demonstration of clinical signs including abdominal pain and the diagnosis of acute pancreatitis. Only then was the pleural exudate tested for an amylase level. It was this study that led to the suspicion of a pancreaticopleural fistula. Surgical treatment is the definitive way to treat this type of fistula [13]. Due to the commonly identified inflammatory infiltration around the pancreas as well as in the retroperitoneal space, the large vessels, and the surrounding organs, surgical interventions can often lead to severe complications, including death. Total excision of the fistula may not be initially possible, and it may be more reasonable to minimize the initial surgery: for example, the first intervention may involve drainage with further staged surgeries planned when the acute inflammation resolves, though this may not always be practical [14]. In the described case, 8 months after the first symptoms and the patient's first hospitalization, the correct diagnosis was made, and the proper treatment was performed. The fistula tract was identified and separated, and consequently, the patient condition improved. Being aware of this rare complication of pancreatitis may help to correctly diagnose future patients who present with intractable, recurrent pleural effusions, even in the absence of a known history of pancreatitis.

## Data Availability

The individual patient's data used to support the findings of this study are available from the corresponding author upon request. Previously reported data are cited at relevant places within the text as References [1–14].
